# Supplementary material for: Intrinsic properties link a network model to zebra finch song
Source: eLife. 2026 Jun 25;13:RP99611. doi: 10.7554/eLife.99611 (PMC13299593; doi:10.7554/eLife.99611)
Supplement: Peer review history

# Editor report: eLife Assessment

**DOI:** 10.7554/eLife.99611.3.sa0

**Authors:** Catherine Emily Carr

**Affiliations:** University of Maryland United States

**Keywords:** Important, Solid

In this manuscript the authors examine correlations between intrinsic electrophysiological properties of HVC neurons projecting to Area X and the temporal structure of the birds' song. The study provides **important** insights into how the structure of vocalization can relate to intrinsic physiological properties of the neurons that are essential for learning the behavior. The evidence supporting the idea that song temporal structure is related to intrinsic physiology is **solid** and this research will be of general interest to researchers in the field and neurophysiologists.

# Referee report: Reviewer #1 (Public Review)

**DOI:** 10.7554/eLife.99611.3.sa1

Summary:

Previous research from the Margoliash laboratory has demonstrated that the intrinsic electrophysiological properties of one class of projection neurons in the song nucleus HVC, HVCX neurons, are similar within birds and differ between birds in a manner that relates to the bird's song. The current study builds on this research by addressing how intrinsic properties may relate to the temporal structure of the bird's song and by developing a computational model for how this can influence sequence propagation of activity within HVC during singing.

First, the authors identify that the duration of the song motif is correlated with the duration of song syllables and particularly the length of harmonic stacks within the song. They next found positive correlations between some of the intrinsic properties, including firing frequency, sag ratio, and rebound excitation area with the duration of the birds' longest harmonic syllable and some other measure of motif duration. These results were extended by examining measures of firing frequency and sag ratio between two groups of birds that were experimentally raised to learn songs that only differed by the addition of a long terminal harmonic stack in one of the groups. Lastly, the authors present an HH-based model elucidating how the timing and magnitude of rebound excitation of HVCX neurons can function to support previously reported physiological network properties of these neurons during singing.

Strengths:

By trying to describe how intrinsic properties (IPs) may relate to the structure of learned behavior and providing a potentially plausible model (see below for more on this) for how differences in IPs can relate to sequence propagation in this neural network, this research is addressing an important and challenging issue. An understanding of how cell types develop IPs and how those IPs relate to the function and output of a network is a fundamental issue. Tackling this in the zebra finch HVC is an elegant approach because it provides a quantifiable and reliable behavior that is explicitly tied to the neurons that the authors are studying. Nonetheless, this is a difficult problem, and kudos to the authors for trying to unravel this.

Correlations between harmonic stack durations and song durations are well-supported and interesting. This provides a new insight that can and will likely be used by other research groups in correlating neuronal activity patterns to song behavior and motif duration. Additionally, correlations between IPs associated with rebound excitation are also well supported in this study.

The HH-model presented is important because it meaningfully relates how high or low rebound excitation can set the integration time window for HVCX neurons. Further, the synaptic connectivity of this model provides at least one plausible way in how this functions to permit the bursting activity of HVCX neurons during singing (and potentially during song playback experiments in sleeping birds). Thus, this model will be useful to the field for understanding how this network activity intersects with 'learned' IPs in an important class of neurons in this circuit.

Comments on revised version:

The authors have adequately addressed my previous concerns.

# Referee report: Reviewer #2 (Public Review)

**DOI:** 10.7554/eLife.99611.3.sa2

Intrinsic properties of a neuron refer to the ion channels that a neuron expresses. These ion channels determine how a neuron responds to its inputs. How intrinsic properties link to behavior remains poorly understood. Medina and Margoliash address this question using the zebra finch, a well-studied songbird. Previous studies from their lab and other labs have shown that the intrinsic properties of adult songbird basal-ganglia projecting premotor neurons, are more similar within a bird than across birds. Across birds, this similarity is related to the extent of similarity in the songs; the more similar the song between two birds, the more similar the intrinsic properties between the neurons of these two birds. Finally, the intrinsic properties of these neurons change over the course of development and are sensitive to intact auditory feedback. However, the song features that relate to these intrinsic properties and the function of the within-bird homogeneity of intrinsic properties are unclear.

In this manuscript, the authors address these two questions by examining the intrinsic properties of basal-ganglia projecting premotor neurons in zebra finch brain slices. Specifically, they focus on the Ih current (as this is related to rhythmic activity in many pattern-generating circuits) and correlate the properties of the Ih current with song features. They find that the sag ratio (a measure of the driving force of the Ih current) and the rebound area (a measure of the post-inhibitory depolarisation) are both correlated with the temporal features of the song. First, they show the presence of correlations between the length of the song motif and the length of the longest syllable (most often a harmonic stack syllable). Based on this, they conclude that longer song motifs are composed of longer syllables. Second, they show that HVCX neurons within a bird have more similar sag ratios and rebound areas than across birds. Third, the mean sag ratio and mean rebound areas across birds were correlated with the duration of the longest harmonic stack within the song. These two results suggest that IPs are correlated with the temporal structure of the song. To further test this, the authors used natural and experimental tutoring procedures to have birds that learned two different types of songs that only differed in length; the longer song had an extra harmonic stack at the end. Using these two sets of birds, the authors find larger sag ratios and higher firing frequencies in birds with longer songs. Fifth, they show that the post-inhibitory rebound area allows neurons to respond to excitatory inputs and produce spikes. Neurons with a larger rebound area have a larger time window for responding to excitatory inputs. Based on this, they speculate that HVCX neurons with larger rebound areas integrate over larger time windows. Finally, they make a network model of HVC and show that one specific model could explain sequence-specific bursting of HVCX neurons.

Strengths:

The question being addressed is an interesting question and the authors use appropriate techniques. The authors find a new temporal structure within the song, specifically, they find that longer songs typically have more syllables and longer syllables. As far as I know, this has not been shown earlier. The authors build on existing literature to suggest that IPs of HVCX neurons are correlated with the temporal structure of songs.

Comments on revised version:

I have read through the revised paper and I also feel that my comments have been addressed.

# Referee report: Reviewer #3 (Public Review)

**DOI:** 10.7554/eLife.99611.3.sa3

It is rare to find systems in neuroscience where a detailed mechanistic link can be made between the biophysical properties of individual neurons and observable behaviors. In this study, Medina and Margoliash examined how the intrinsic physiological properties of a subclass of neurons in HVC, the main nucleus orchestrating the production of birdsong, might have an effect on the temporal structure of a song. This builds on prior work from this lab demonstrating that intrinsic properties of these neurons are highly consistent within individual animals and more similar between animals with similar songs, by identifying specific acoustic features of the song that covary with intrinsic properties and by setting forth a detailed biophysical network model to explain the relationship.

The main experimental finding is that excitability, hyperpolarization-evoked sag, and rebound depolarization are correlated with song duration and the duration of long harmonic elements. This motivates the hypothesis that rebound depolarization acts as a coincidence detector for the offset of inhibition associated with the previous song element and excitation associated with the start of the next element, with the delay and other characteristics of the window determined primarily by Ih. The idea is then that the temporal sensitivity of coincidence detection, which is common to all HVCx neurons, sets a global tempo that relates to the temporal characteristics of a song. This model is supported by some experimental data showing variation in the temporal integration of rebound spiking and by a Hodgkin-Huxley-based computational model that demonstrates proof of principle, including the emergence of a narrow (~50 ms) post-inhibitory window when excitatory input from other principal neurons can effectively evoke spiking.

Overall, the data are convincing and the model is compelling. The manuscript plays to the strengths of zebra finch song learning and the well-characterized microcircuitry and network dynamics of HVC. Of particular note, the design for the electrophysiology experiments employed both a correlational approach exploiting the natural variation in zebra finch song and a more controlled approach comparing birds that were tutored to produce songs that differed primarily along a single acoustical dimension. The modeling is based on Hodgkin-Huxley ionic conductances that have been pharmacologically validated, and the connections and functional properties of the network are consistent with prior work. This makes for a level of mechanistic detail that will likely be fruitful for future work.

Comments on revised version:

I read through everything and I also feel that my comments have been adequately addressed.

# Author comment: Author response

**DOI:** 10.7554/eLife.99611.3.sa4

**Authors:** Nelson Medina, Dan Margoliash

**Affiliations:** University of Chicago Chicago United States; University of Chicago Chicago United States

The following is the authors’ response to the original reviews.

**Reviewer #1(Public Review):**

The correlation between rebound excitation and song structure (e.g., harmonic stack duration) may depend on outliers, such as birds with harmonic stacks >150ms.

If in wild zebra finch, or even if in domesticated zebra finch including our birds and the birds from the other labs that we evaluated, the distribution of durations of longest harmonic stacks has a long tail, it is not apparent that birds with long duration harmonic stacks are properly considered as outliers. Examining the distribution of motif durations (a less derived statistic) in 33 birds (Fig. 2C) does not support the idea that birds with longer duration songs are outliers. Thus, we view the reviewer question as addressing whether there are different mechanisms operating in birds with long harmonic stacks than for other birds. Unfortunately, the numbers of long-duration harmonic stack birds are too small to give confidence in any statistical analysis of that group. Thus, we limited our re-analysis to the data excluding birds with harmonic stacks >150ms (which is arbitrary), examining how these birds influence our conclusions. We conclude that the influence of the excluded birds on the overall result is modest. The updated results are presented in Supplemental Figure 6, and the Results section has been revised to state:

“We found that while some of the p values increased above 0.05 (p = 0.058 for rebound area vs. longest harmonic stack and p = 0.082 for sag ratio and longest harmonic stack), it remained significant for firing frequency and longest stack (Pearson’s R, p = 0.0017) and for sag ratio and motif duration (p = 0.024). However, when sag ratio was compared against the duration of the motif excluding the longest harmonic stack, there was no relationship (p = 0.85).”

There is a disconnect between the physiological measurements and the HH model presented.

We acknowledge that addressing this limitation would involve additional experimental and modeling assumptions. Rather than overextending our interpretations, we have clarified the limitations of the current study in the Discussion:

“While this HH model provides a plausible framework for linking intrinsic properties to sequence propagation, it does not fully account for the observed relationship between IPs and song structure. A principal limitation constraining the current model is the absence of information for the same neurons combining characterization of both IPs and network activity during singing (or song playback), when HVC_X_ express activity related to song features. Addressing this gap would requires additional and challenging experiments and is beyond the scope of this study.”

Although disynaptic inhibition between HVC_X_ neurons and between HVC_RA_ and HVC_X_ neurons is well established, I am not aware of any data indicating direct synaptic connections between HVC_X_ neurons.

This is an important theoretical point about the reliance of the intervaldetecting network model on HVC_X_ neurons and about how the model would change if many of the HVC_X_ were swapped for HVC_RA_ neurons. Connections between HVC_RA_ neurons to HVC_X_ neurons are established, whereas there is relative paucity of evidence for HVC_X_ to HVC_X_ connectivity. This is based on work from Prather and Mooney, 2005 (among others) which performed paired sharp electrode recordings to characterized connections in HVC. This work found very few HVC_X_ - HVC_X_ connections. However, if connected HVC_X_ neurons are physically more distant from each other than are connected HVC_RA_ – HVC_X_ neurons, they would more likely be missed in blind paired recordings. Using different approaches, recent results from the Roberts lab (Trusel et al.,eLife, 2025) supports the existence of robust HVC_X_ - HVC_X_ connections.

**Reviewer #2(Public Review):**

The interpretation of p-values is rigid, and near-significant results (e.g., p = 0.06) are dismissed without discussion.

We revised the text to reflect a more nuanced and consistent interpretation of p-values and updated the reporting to include exact values. For example, the Results section now states:

"Nonetheless, the longest syllable duration was not significantly correlated with the average sag ratio for each bird (Pearson’s R: R^2^ = 0.12, p = 0.065, Supplemental Fig. 2, top left panel), though it is trending toward significance (see Discussion)”

The conclusion that harmonic stacks influence intrinsic properties lacks necessary controls.

We have attempted to further clarify that harmonic stacks were used as a representative feature of temporal song structure rather than a unique determinant of intrinsic properties. The Discussion now states:

“Although harmonic stacks provide a useful test case for studying temporal integration, our findings suggest that IPs are broadly linked to song duration and structure, rather than specific syllable types. This is also consistent with prior results that found all HVC_X_ ion currents that were modeled were influenced by song learning[31].”

The relationship between rebound area and experimentally tutored birds was not fully explored.

We expanded the analysis to include rebound area in instrumentally tutored birds, which has now been incorporated into Figure 4C. These additional analyses also robustly support our hypotheses. The Results section has been updated to state:

“We then evaluated the IPs of HVC_X_ in the birds from the two groups. HVC_X_ neurons from birds who sang unmodified songs (N = 5 birds, 31 neurons), which had shorter harmonic stacks and shorter overall duration, had lower sag ratios (Mann-Whitney: p = 0.025), firing frequency (Mann-Whitney, p = 0.0051) and rebound area (Mann-Whitney: p = 0.0003)”

**Reviewer #3 (Public Review):**

Limited data supports the claim that intrinsic properties influence temporal integration windows.

While we agree that further data could strengthen this claim, we show that this can happen in principle (Figure 5) but believe that the appropriate experiment to test this requires further experiments in-vivo. We emphasize in the Discussion:

“Our findings suggest that post-inhibitory rebound excitation in HVC_X_ could expand temporal integration. Ultimately, experiments combining in vitro with in vivo recordings can directly quantify this effect. We hope our results motivate such experiments.”

Technical Corrections

(1) Fixed typographical errors (e.g., Line 177: corrected "r2 = 4" to "r2 = 0.4").

(2) Revised figure legends for clarity (e.g., Figure 4E now includes tutoring design details).

(3) Updated methods to specify how motifs were defined and measured.

Revised Figures

Figure 4: Updated to include analysis of rebound area in instrumentally tutored birds, reflecting the relationship between experimental tutoring and intrinsic properties.

Supplemental Figure 6: Correlation analysis excluding outliers
